# The Application of Machine Learning Algorithms to Predict HIV Testing in Repeated Adult Population–Based Surveys in South Africa: Protocol for a Multiwave Cross-Sectional Analysis

**DOI:** 10.2196/59916

**Published:** 2025-01-27

**Authors:** Musa Jaiteh, Edith Phalane, Yegnanew A Shiferaw, Refilwe Nancy Phaswana-Mafuya

**Affiliations:** 1 South African Medical Research Council/University of Johannesburg Pan African Centre for Epidemics Research Extramural Unit Faculty of Health Sciences University of Johannesburg Johannesburg South Africa; 2 Department of Statistics Faculty of Science University of Johannesburg Johannesburg South Africa

**Keywords:** predictive modelling, testing, support vector machines, random forest, supervised machine learning, decision trees, adult, population-based, South Africa, protocol, HIV/AIDS, HIV testing, retrospective analysis, cross-sectional survey, chi-square test, logistic regression, public health, epidemiology, infectious disease

## Abstract

**Background:**

HIV testing is the cornerstone of HIV prevention and a pivotal step in realizing the Joint United Nations Program on HIV/AIDS (UNAIDS) goal of ending AIDS by 2030. Despite the availability of relevant survey data, there exists a research gap in using machine learning (ML) to analyze and predict HIV testing among adults in South Africa. Further investigation is needed to bridge this knowledge gap and inform evidence-based interventions to improve HIV testing.

**Objective:**

This study aims to determine consistent predictors of HIV testing by applying supervised ML algorithms in repeated adult population-based surveys in South Africa.

**Methods:**

A retrospective analysis of multiwave cross-sectional survey data will be conducted to determine the predictors of HIV testing among South African adults aged 18 years and older. A supervised ML technique will be applied across the five cycles of the South African National HIV Prevalence, Incidence, Behavior, and Communication Survey (SABSSM) surveys. The Human Science Research Council (HSRC) conducted the SABSSM surveys in 2002, 2005, 2008, 2012, and 2017. The available SABSSM datasets will be imported to RStudio (version 4.3.2; Posit Software, PBC) to clean and remove outliers. A chi-square test will be conducted to select important predictors of HIV testing. Each dataset will be split into 80% training and 20% test samples. Logistic regression, support vector machines, random forests, and decision trees will be used. A cross-validation technique will be used to divide the training sample into k-folds, including a validation set, and models will be trained on each fold. The models’ performance will be evaluated on the validation set using evaluation metrics such as accuracy, precision, recall, *F*_1_-score, area under curve-receiver operating characteristics, and confusion matrix.

**Results:**

The SABSSM datasets are open access datasets available on the HSRC database. Ethics approval for this study was obtained from the University of Johannesburg Research and Ethics Committee on April 23, 2024 (REC-2725-2024). The authors were given access to all five SABSSM datasets by the HSRC on August 20, 2024. The datasets were explored to identify the independent variables likely influencing HIV testing uptake. The findings of this study will determine consistent variables predicting HIV testing uptake among the South African adult population over the course of 20 years. Furthermore, this study will evaluate and compare the performance metrics of the 4 different ML algorithms, and the best model will be used to develop an HIV testing predictive model.

**Conclusions:**

This study will contribute to existing knowledge and deepen understanding of factors linked to HIV testing beyond traditional methods. Consequently, the findings would inform evidence-based policy recommendations that can guide policy makers to formulate more effective and targeted public health approaches toward strengthening HIV testing.

**International Registered Report Identifier (IRRID):**

DERR1-10.2196/59916

## Introduction

HIV/AIDS remains a public health threat, affecting 39 million people globally, of whom 25.6 million are within the Sub-Saharan African region [1]. More than half of the global HIV prevalence is concentrated in East and Southern Africa, according to the World Health Organization and the Joint United Nations Program on HIV/AIDS (UNAIDS) [1,2]. HIV testing is a crucial component of the HIV prevention and care continuum [3] and a pivotal step in realizing the UNAIDS’s goal of ending AIDS by 2030 [4]. It aims to diagnose and minimize HIV transmission rates while enabling early and effective treatment. Rolling out HIV testing coverage and accessibility to populations with low HIV testing prevalence could assist in improving HIV epidemic control and elimination efforts [3-7].

South Africa has made significant progress toward the UNAIDS 2030 target, ensuring 90% of people living with HIV are aware of their status by the end of 2022 [8,9]. However, the country remains behind schedule as the 90% attainment was set for 2020. Furthermore, there still exist numerous barriers to the uptake of HIV testing [10]. According to Simbayi et al [10], lack of HIV testing is one of the key drivers of HIV infections among the South African adult population.

Previous studies have identified sociodemographic factors, sociobehavioral factors, socioeconomic factors, sexual behaviors, knowledge, attitudes, and perceptions about HIV that could influence an individual’s decision to undergo HIV testing [6,7,11,12]. The uptake of HIV testing was found to be very low (34.9%) among Ethiopian youth due to regional variations [11]. The study revealed that being a male, following a protestant religion or orthodox religion negatively influenced the uptake of HIV testing [11]. The same study [11] noted that Ethiopian youth might undergo HIV testing if they have a good knowledge of HIV, engage in risky sexual behavior, are married, are rich, and have access to media. A retrospective analysis of the 2017 South African National HIV Prevalence, Incidence, Behavior, and Communication Survey (SABSSM) survey by Jooste et al [12] highlighted geographic variations by districts and sex in HIV testing among individuals aged 15 years and older. The districts with the lowest HIV testing were uMkhanyakude (54.7%) and Ugu (61.4%) in KwaZulu-Natal and Vhembe (61%) in Limpopo [12]. On the other hand, the uptake of HIV testing was higher among women than men in most of the districts [12]. Probably stemming from the common perception that women are at the highest risk of HIV, numerous studies substantiated a greater hesitancy in HIV testing uptake among men compared to women [11,12]. Socioeconomic factors such as rural residence, low education, household income, and high alcohol consumption have also been associated with low HIV testing uptake [13].

Traditional statistical and testing approaches have been widely used to scale up HIV testing interventions worldwide; while they are effective, they can be subject to certain limitations, such as accuracy, precision, and privacy issues [14]. In contrast, machine learning (ML) models are designed to address these limitations, as they can analyze complex datasets to uncover latent variables associated with HIV testing beyond the reach of conventional methodologies [15,16]. ML refers to the use and development of computer systems that learn and adapt without following explicit instructions, and algorithms are used to analyze and draw conclusions from data patterns [17,18]. The algorithms rely on domain knowledge of the data to develop features that make them work [18]. Various disciplines, including health care, use 3 major types of ML, namely, supervised machine learning (SML), unsupervised machine learning, and reinforcement learning [17-19].

A growing number of studies are developing predictive models to classify HIV high-risk populations for improved HIV testing in a resource-efficient manner [20-24]. Ovalle et al [25] applied support vector machines (SVM), random forest, and logistic regression within a Gay Social Networking Analysis Program, an intelligent web-based health-promotion intervention framework. This study explored the link between social media messages and participants’ offline sexual health and substance use outcomes, deriving HIV risk scores for targeted HIV testing [25]. Various SML algorithms such as logistic regression, SVM, random forest, and XGBoost were used to accurately predict HIV risk in South Africa, in which age, being a female, and condom use were significantly associated with HIV positivity [26].

Evidently, ML techniques facilitate the development of economically viable HIV testing methods that offer optimal value for resources invested [16]. Importantly [16], asserts that HIV risk prediction with ML techniques could increase the HIV-diagnosed fraction to 96.5% by 2030, compared to the current traditional method projection of a diagnosed fraction of 93.8% by the same year in South Africa.

Although a significant amount of survey data is available on the factors associated with HIV testing in South Africa, there has been limited research on using ML approaches to analyze and forecast these factors. Thus, it is against this backdrop that this study is pioneering in using four SML algorithms (logistic regression, random forest, SVM, and decision trees) across all five cycles of the SABSSM surveys to provide new insights into the determinants of HIV-testing behavior in South Africa. The primary aim of this study is to determine consistent predictors of HIV testing and to compare the performance of the four SML models using repeated adult population-based surveys in South Africa to develop an evidence-based predictive model to enhance HIV testing.

## Methods

### Study Design

A retrospective analysis of data from multiple cross-sectional surveys will be used to predict factors associated with HIV testing across the five cycles of the SABSSM survey using SML algorithms. The analysis will involve four SML algorithms in developing an HIV testing predictive model for the South African adult population. This protocol followed several guidelines and checklists, such as the STROBE (Strengthening the Reporting of Observational Study in Epidemiology) checklist [27] (this can be found in Multimedia Appendix 1), the updated guidance for reporting clinical prediction models that use regression or ML [28] (this can be found in Multimedia Appendix 2), and the development and validation of observational and qualitative study protocol reporting checklists for novice researchers (ObsQual checklist) [29].

### Data Source and Population

This secondary data analysis will make use of the 5 cycles of the SABSSM surveys conducted by the South African Human Science Research Council (HSRC). The SABSSM surveys are a series of nationally representative surveys conducted periodically in South Africa to assess the prevalence, incidence, behaviors, and communication related to HIV/AIDS [10]. The first SABSSM survey was conducted in 2002 with 9963 individuals interviewed, followed by SABSSM 2005 (n=23,275), SABSSM 2008 (n=20,826), SABSSM 2012 (n=38,431), and SABSSM 2017 (n=36,609) being the fifth survey [10,30-33]. All five cycles of the SABSSM datasets were made available to the research team on August 20, 2024, after filling and submitting the digital request form via the HSRC website [34]. This study’s analysis will focus on adults aged 18 years and older, using data from the five cycles of the SABSSM survey—2002, 2005, 2008, 2012, and 2017. The final sample size for this study will be determined after cleaning and removing outliers from the datasets.

### Inclusion and Exclusion Criteria

The SABSSM dataset is a combination of children’s, adolescents’, and adults’ data. This analysis will only include male and female individuals aged 18 years and older from the SABSSM survey. Data outside the defined age bracket from the SABSSM survey data will be excluded from this analysis.

### Study Variables

The outcome variable for this study will be HIV testing. The exposure variables are sociodemographic (age, sex, ethnicity or race, education level, marital status, employment, and rural and urban residence), socioeconomic (household income, financial status, and access and usage of health care facilities), sexual behaviors (history of sexually transmitted infections, knowledge of HIV transmission, number of sexual partners, and condom use), HIV knowledge and awareness (knowledge toward HIV prevention, testing and treatment services, and knowledge of HIV status), and perceptions and attitudes (attitudes toward HIV testing, perceived risk of infection, stigma, and discrimination related to HIV). Table 1 describes a few selected variables that are consistent across all the five SABSSM survey datasets. The complete list of the dependent and independent variables extracted from 2002, 2005, 2008, 2012, and 2017 SABSSM survey datasets can be found in Multimedia Appendix 3 (SABSSM 2002 variables), Multimedia Appendix 4 (SABSSM 2005 variables), Multimedia Appendix 5 (SABSSM 2008 variables), Multimedia Appendix 6 (SABSSM 2012 variables), and Multimedia Appendix 7 (SABSSM 2017 variables).

**Table 1:** Summary of variable and variable definitions from the SABSSM^a^ dataset^b^.

Variable name and variables					Variable descriptions		Variable recode
**Outcome variable (HIV testing)**							
	**HIV testing**						
		Ever had an HIV test			Yes No No response		Yes No
**Exposure variables**							
	**Sociodemographic**						
		**Age**					
			Respondent’s age in years	Integer		<20 20-24 25-29 30-34 35-39 40-44 45-49 ≥50	
		**Sex**					
			Sex of the respondent	Male Female		Male Female	
		**Geotype**					
			Geographical location	Urban formal Urban informal Rural formal Rural informal		Urban Rural	
		**Race**					
			Race	African White Colored Indian or Asian Other		African Colored Indian White	
		**Highest level of education**					
			Respondent’s highest level of education obtained	No schooling Up to Std^c^ 1/Gr^d^ 3/ABET^e^ 1 Std 2-Std 3/Gr 4-Gr 5/ABET 2 Std 4-Std 5/ Gr 6-Gr 7/ABET 3 Std 6-Std 7/Gr 8-Gr 9/ABET 4 Std 8/Gr 10/NTC^f^ 1 Std 9/Gr 11/NTC 2 Std 10/Gr 12/Matric/NTC 3 Certificate or diploma with Gr 12 Bachelors degree Postgraduate degree (HDE/Hons/Masters/PhD)		No schooling Primary Secondary Tertiary	
	**Sociocultural**						
		**Age at first marriage**					
			How old was the respondent when married for the first time	Integer		<18 18-23 24-29 30-35 >35	
		**Male circumcision status**					
			Whether the male respondent is circumcised	Yes No		Yes No	
**Independent variable (socioeconomic)**							
	**Employment status**						
		Employment status			Unemployed Sick or disabled and unable to work Student or pupil or learner Employed or self-employed Other		Unemployed Sick or disabled and unable to work Student or pupil or learner Employed or self-employed Other
**Independent variable (sexual history, sexual behavior, and lifestyle)**							
	**Ever had sexual intercourse**						
		Whether the respondent ever had sexual intercourse			Yes No No Response		Yes No
	**Number of sexual partners in a lifetime**						
		Number of people the respondent had sexual intercourse within a lifetime			Integers		1 person 2-5 >5
	**Condom use decision**						
		Who suggested using a condom? The second most recent person			Yourself Your partner Mutual agreement		Yourself Your partner Mutual agreement
**Independent variable (health status or pre-existing medical conditions or disabilities or stigma or violence)**							
	**General well-being**						
		In general, would you say that your health is excellent, good, fair, or poor?			Excellent Good Fair Poor		Excellent Good Fair Poor
**Independent variable (knowledge, awareness, and perception of HIV/AIDS)**							
	**Knowledge of HIV/AIDS**						
		Can AIDS be cured?			Yes No I don’t know		Yes No I don’t know

^a^SABSSM: South African National HIV Prevalence, Incidence, Behavior, and Communication Survey.

^b^Only a few selected variables that are common across the five SABSSM surveys were included in Table 1. Please see Multimedia Appendices 1-5 for the complete list of dependent and independent variables.

^c^Std: standard.

^d^Gr: grade.

^e^ABET: adult basic education training.

^f^NTC: National Technical Certificate.

### Data Extraction

A data extraction tool (Table 2) with headings such as study year, sample size, study population, age group study settings, and main variables was used to describe the characteristics of the available datasets of the SABSSM surveys. Each dataset was examined, and key variables that could influence HIV testing were identified using additional tools (Table 1, Multimedia Appendix 3 [SABSSM 2002 variables], Multimedia Appendix 4 [SABSSM 2005 variables], Multimedia Appendix 5 [SABSSM 2008 variables], Multimedia Appendix 6 [SABSSM 2012 variables], and Multimedia Appendix 7 [SABSSM 2017 variables]).

**Table 2:** Characteristics of the five SABSSM^a^ survey datasets^b,c^.

Study name and year	Sample size, n			Prevalence of HIV testing (reported), %
	Calculated	Individual Interview Response		
SABSSM 2002 [30]	14,450	9963	21.4	
SABSSM 2005 [31]	24,236	23,275	30.5	
SABSSM 2008 [32]	23,369	20,826	50.8	
SABSSM 2012 [33]	42,950	38,431	65.5	
SABSSM 2017 [10]	39,132	36,609	75.2	

^a^SABSSM: South African National HIV Prevalence, Incidence, Behavior, and Communication Survey.

^b^The study is focused on the general population of South African adults aged 18 years and older.

^c^Study variables were extracted and redefined to guide the data cleaning and preprocessing, as detailed in the data preprocessing section.

### Data Preprocessing and Feature Selection

The SABSSM survey datasets are open access data available upon request through the HSRC website [34]. All five cycles of the SABSSM datasets were accessed on August 20, 2024. We have explored them to understand the patterns and the nature of all the variables needed to develop an HIV testing predictive model. For this analysis, RStudio (version 4.3.2; Posit Software, PBC) will be used. The research team will first clean and process the available data to handle outliers and missing values and identify relevant dependent and independent variables in the five cycles of the SABSSM survey to be included in the predictive modeling. This will be followed by feature selection to eliminate redundant and irrelevant variables. Numerical features will be standardized to ensure consistency scaling, and the features will be analyzed to categorize those that will most likely influence HIV testing. We will conduct a chi-square test between the dependent variable (reported HIV testing status) and the independent variables (Table 1). Variables (features) with *P*<.05 will be considered important predictors and will be retained in the final model for predicting HIV testing status. Potential confounders and redundant variables will also be addressed during the feature selection, as detailed in the risk for bias assessment section. One-hot encoding will be used to encode the important HIV testing predictors based on the information available on the SABSSM surveys. One-hot encoding is the process of transforming categorical variables into binary variables, represented by values of 0 and 1, enabling their usage by ML algorithms [35]. This procedure will be accomplished by an R package known as “dplyr” [36].

### Algorithms

This study will use four SML algorithms that are widely used in the field of health care, namely, logistic regression, decision trees, random forests, and SVM [37]. Logistic regression is a statistical method commonly used in ML for classification and predictive analytics tasks [38]. It solves classification problems by assigning multiple observations to a discrete set of categories or cases [38]. Another classifier that will be used in this analysis is SVM, a group of SML algorithms that can be applied to classification or regression [39]. The SVM can act as nonprobabilistic binary linear classifiers, depending on their configuration. For instance, a linear kernel function would make an SVM perform as a binary linear classifier. Moreover, SVM can also handle nonlinear classification tasks [39]. Similarly, a decision tree is also an SML classifier that categorizes or predicts depending on prior sets of questions [40]. According to Jo [19], a decision tree resembles a tree that classifies based on the branches of its root nodes. Random forest is also a very popular SML algorithm [22]. It is a robust SML algorithm well-suited for classification tasks involving complicated relationships between different features.

### Training and Model Validation

After the preprocessing and feature selection, each cleaned dataset will be split into 80% training and 20% testing samples. The 80/20 rule is the most used splitting ratio, and anecdotal evidence has supported it as being an effectively valid rule [41]. Training and validation will be done to set a probability threshold to predict those factors associated with HIV testing. Four standard SML algorithms will be applied to the training sample, such as logistic regression, decision trees, random forests, and SVM [37]. A cross-validation technique will be used, in which the training sample will be divided into k-folds, including a validation set, and models will be trained on each fold. Usually, k is taken to be 5 or 10, but may differ depending on the dataset size [42]. This study will use either a 5-fold or 10-fold cross-validation technique, depending on the compatibility with the available datasets. The models’ performance will be evaluated on the validation set using the following evaluation metrics: accuracy, precision, recall, *F*_1_-score, area under curve-receiver operating characteristics, and confusion matrix.

### Evaluation and Algorithms Selection for Predicting HIV Testing

**Figure 1:**
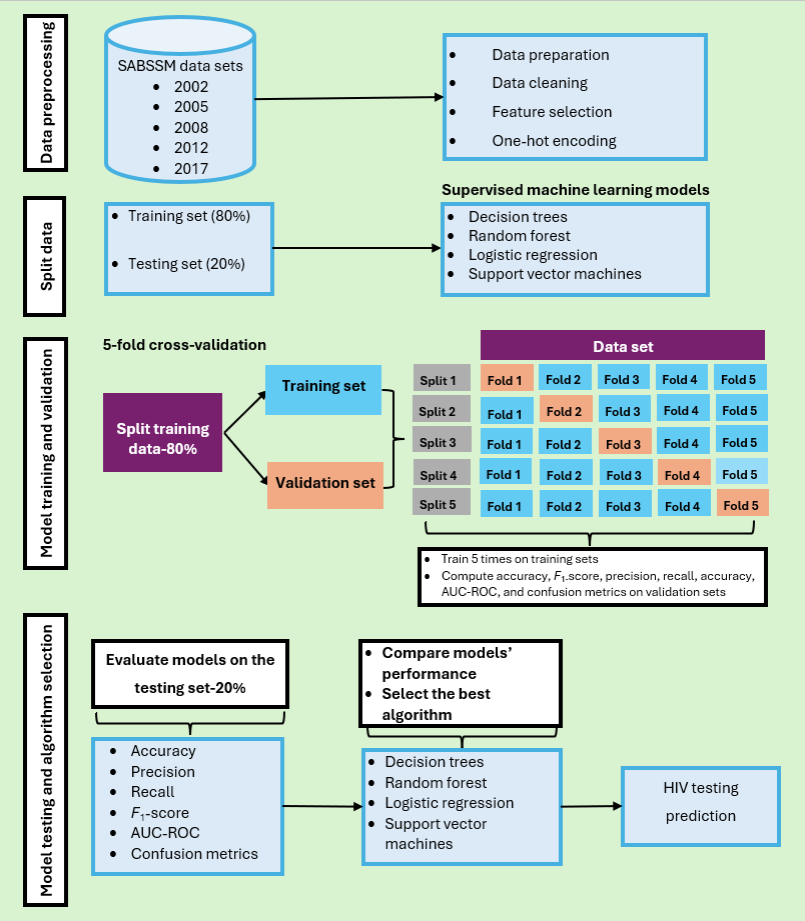
Data preprocessing and HIV testing prediction modeling (created using PowerPoint [version 16.0.1; Microsoft Corp] diagram flow and concept guided by Mutai et al [21], Chingombe et al [38] and Chingombe et al [43]. AUC-ROC: area under curve-receiver operating characteristics; SABSSM: South African National HIV Prevalence, Incidence, Behavior, and Communication Survey.

Model evaluation involves selecting the best-performing model by providing an unbiased estimate of how well the models generalize on new or unseen data [18]. Upon completing the model development, the performance of the four SML models will be assessed on the testing sample using standardized performance evaluation metrics, including accuracy, precision, recall, *F*_1_-score, area under curve-receiver operating characteristics, and confusion matrix [43]. This procedure will assess the performance of logistic regression, SMV, random forest, and decision trees in order to select the best-performing models in predicting HIV testing status using the five cycles of the SABSSM datasets. Figure 1 [21,43] depicts the data processing stages, HIV testing predictive models to be used, and performance evaluation criteria.

### Risk for Bias Assessment

Since the proposed study will analyze secondary data from multiple cross-sectional data, it is pertinent to assess and mitigate potential bias to ensure the validity and reliability of the results. The Joanna Briggs Institute Critical Appraisal Checklist for Analytical Cross-Sectional Studies (this can be found in the Multimedia Appendix 8) will be used to mitigate the risk of bias in this study. At first, the researchers reviewed published reports of the five SABSSM surveys to understand the study designs, study population, sample biases, study variables, and the data collection methods used in each survey by the HSRC. This process guided the development of the study design, eligibility criteria, and variable selection for this study. As of August 20, 2024, the authors have started exploring the SABSSM datasets to clean the data and treat missing values. Imbalanced data occurs when one class has significantly more observations than the other, leading algorithms to be biased toward the majority class. In such cases, the researchers will use various techniques to address this and ensure fair model performance. Potential confounders of HIV testing will be identified based on consultations with the literature, as well as the use of statistical measures. Additionally, a variance inflation factor will be used to detect multicollinearity and remove redundant variables associated with HIV testing. This process will also inform the selection of features to be used for the HIV testing predictive modeling.

### Data Management

This predictive modeling will use the SABSSM datasets sourced from the HSRC, with no personal information collected from the study participants. Following the data preprocessing and analysis, the preliminary results will be maintained securely using a memory stick that requires an access code. Only the researcher and the research team will be able to review these findings. The results to be published will not contain any identifying information. The electronic data will be stored in the Boloka data repository, password-protected with username access. The Boloka project aims to harness big heterogeneous data to evaluate the impact of HIV responses among key populations in generalized epidemic settings in Sub-Saharan Africa. All the data used in this study will be managed in compliance with the Protection of Public Information Act. The data generated from this study will be securely stored in the Boloka data repository for five years following the study’s completion. It is important to note that the SABSSM datasets are governed by an End User License. As per the data-sharing conditions stipulated by the HSRC, the study’s data cannot be duplicated, reshared, or sold without prior approval from the rights holder [34].

### Ethical Considerations

This study protocol forms part of a doctoral study at the University of Johannesburg (UJ) by the first author (MJ), titled, “Integration of machine learning algorithms to predict HIV testing associations using repeated cross-sectional survey data in an adult South African population: an HIV testing predictive model.” The study protocol was approved by the UJ Research and Ethics Committee on April 23, 2024; ethics approval REC-2725-2024 (this can be found in Multimedia Appendix 9). Further, the doctoral study falls under an umbrella project funded by the South African Medical Research Council under the South African Medical Research Council/UJ Pan African Centre for Epidemics Research Extramural Unit, titled: “Harnessing big heterogeneous data to evaluate the potential impact on HIV responses among the key populations in generalized epidemic settings in Sub-Saharan Africa” (ethics approval: REC-1504-2022). Since this study will analyze secondary data, a waiver of informed consent for secondary data use was obtained from the UJ Research and Ethics Committee (this can be found in Multimedia Appendix 9). According to Shisana and Simbayi [30], Shisana et al [31-33], and Simbayi et al [10], all the SABSSM surveys have been approved by the HSRC REC, and studies were conducted in accordance with international ethical standards, as well as the South African Children ACT 2007. Additionally, the SABSSM research team ensured that participants obtained voluntary informed consent. The authors of this study received the SABSSM datasets without any identifying information, and no further efforts will be made to reidentify the study participants during the analysis. The SABSSM datasets are open access data governed by an End User License that cannot be duplicated, reshared, or sold without prior approval from the rights holder (HSRC) [34]. This study will adhere to these data-sharing conditions and all the ethical standards governing the use of secondary data of human participants.

## Results

The authors were given access to the SABSSM datasets for this retrospective analysis by the HSRC on August 20, 2024. All 5 datasets were downloaded from the HSCRC database and explored to identify the independent variables likely to influence HIV testing uptake as of September 30, 2024. The selected dependent (HIV testing status) and dependent variables (age, sex, province, marital status, knowing a place to test for HIV, male circumcision, condom use, sex debut, knowledge and perception of HIV, STI symptoms, visit a doctor, and contraceptive use) were recorded for each SABSSM dataset as similar to Table 1 (complete list of variables can be found in Multimedia Appendix 3 [SABSSM 2002 variables], Multimedia Appendix 4 [SABSSM 2005 variables], Multimedia Appendix 5 [SABSSM 2008 variables], Multimedia Appendix 6 [SABSSM 2012 variables], and Multimedia Appendix 7 [SABSSM 2017 variables]). The preprocessing and feature selection phase described in the methods section will determine which variables are to be used for the HIV testing predictive modeling. This process is expected to be completed by November 30, 2024.

The results of this study will be available by January 31, 2025, and each step of the analysis will be visualized and presented in tables and graphs. The anticipated date of publication for the proposed study is June 2025.

## Discussion

### Principal Findings

The proposed study seeks to identify intricate patterns and relationships among various sociodemographic, behavioral, and contextual variables that contribute to individuals’ decisions to undergo HIV testing in South Africa. Since the first SABSSM survey in 2002, varying rates of HIV testing among South Africans have been reported by the HSRC, showing increased trends in each cycle until 2017 (Table 2). By applying SML learning techniques across the five cycles of the SABSSM survey datasets, the findings of this study will ascertain consistent variables predicting HIV testing uptake among the South African adult population over the 20-year period. Furthermore, the study will evaluate and compare the performance metrics of the four different SML algorithms, and the best model will be used to develop an evidence-based predictive model to enhance HIV testing among the South African adult population.

The emergence of ML applications in health care has demonstrated significant progress. However, predictive modeling in the context of HIV testing is relatively low in developing countries due to challenges such as usability issues, lack of expertise, and insufficient health care data [44]. Steadily, research institutions within specific African countries are increasingly expressing interest in this field.

A study conducted in Tanzania applied SML algorithms, including XGBost and random forest, to develop a model aimed at enhancing HIV index testing, with the recommendation that other health facilities use their model to simplify their work [18]. The study found that knowledge of HIV, age, sex, and marital were significantly associated with HIV index testing [18]. Likewise, a South African study by Majam et al [26] confirms that SML models can be built using digital surveys from low- and middle-income countries to predict high HIV groups for enhanced HIV testing. The models predicted, with over 80% accuracy, that female individuals hesitant to report condom use, and those who had not undergone HIV tests within the past year were at the highest risk of HIV [26]. In addition, an SML technique was used to assess the sociobehavioral predictors of HIV from Demographic and Health Survey data in 10 East and Southern African countries. According to the study, seven male and five female individuals would need to be tested to find one HIV-positive individual [37]. It is also important to consider HIV testing preferences for individuals undergoing HIV testing. Confidentiality, distance from the testing center, and method of data collection were identified as factors determining individual HIV testing preferences in South Africa [45].

The proposed study will expand on the existing knowledge of HIV testing predictors through the application of SML algorithms. Our choice of using SML is based on their heightened predictive accuracy, interpretability, efficiency, and adaptability. The proposed study aims to develop a predictive model to forecast HIV testing associations using repeated cross-sectional survey data in an adult South African population. The developed evidence-based model would assist relevant stakeholders in devising targeting HIV testing interventions in striving toward the UNAIDS 2030 goal of eradicating AIDS in South Africa.

### Strengths and Limitations

To the best of our knowledge, this will be the first study to conduct HIV testing predictive modeling using all 5 cycles of the SABSSM surveys by applying SML algorithms. The large volume of data will be crucial for the generalizability of the developed models, as well as the major findings. It is also important to mention that the research involves a multidisciplinary team of experts across public health, epidemiology, biostatistics, and data science. The well-equipped team will devise a comprehensive methodological approach to developing an adaptable HIV testing predictive model.

However, this study is expected to face some limitations as the SABSSM surveys may contain missing values; proper imputation techniques will be used to address this issue. Since some ML models are not well developed, causing spatial and temporal variations, this could also be a limitation in our proposed study. Furthermore, because the analysis focuses on secondary datasets gathered using cross-sectional designs, the researchers are aware of potential limitations such as biases and lack of control over the variables included. The Joanna Briggs Institute Critical Appraisal Checklist will be used to mitigate the risk of bias in this study.

### Conclusions

This study will contribute to knowledge and deepen understanding of factors linked to HIV testing beyond traditional methods. Consequently, the findings would inform evidence-based policy recommendations that can guide policy makers to formulate more effective and targeted public health approaches toward strengthening HIV testing in South Africa.

## Appendix

Multimedia Appendix 1 STROBE (Strengthening the Reporting of Observational Study in Epidemiology) checklist.

Multimedia Appendix 2 TRIPOD+AI (Transparent Reporting of a multivariable prediction model of Individual Prognosis Or Diagnosis–Artificial Intelligence) checklist.

Multimedia Appendix 3 SABSSM (South African National HIV Prevalence, Incidence, Behavior, and Communication Survey) 2002 variables.

Multimedia Appendix 4 SABSSM (South African National HIV Prevalence, Incidence, Behavior, and Communication Survey) 2005 variables.

Multimedia Appendix 5 SABSSM (South African National HIV Prevalence, Incidence, Behavior, and Communication Survey) 2008 variables.

Multimedia Appendix 6 SABSSM (South African National HIV Prevalence, Incidence, Behavior, and Communication Survey) 2012 variables.

Multimedia Appendix 7 SABSSM (South African National HIV Prevalence, Incidence, Behavior, and Communication Survey) 2017 variables.

Multimedia Appendix 8 JBI (Joanna Briggs Institute) checklist.

Multimedia Appendix 9 Ethics Approval Letter.

## Abbreviations

HSRC: Human Science Resource Council
ML: machine learning
SABSSM: South African National HIV Prevalence, Incidence, Behavior, and Communication Survey
SML: supervised machine learning
STROBE: Strengthening the Reporting of Observational Study in Epidemiology
SVM: support vector machine
UJ: University of Johannesburg
UNAIDS: Joint United Nations Program on HIV/AIDS

## Data Availability

The datasets to be analyzed in the proposed study are not publicly available due to the data-sharing conditions of the Human Science Research Council (HSRC; prohibition of the duplication, resharing, or selling of data without prior approval from the rights holder) but are available from the HSRC on reasonable request [32].
