# Supplementary material for: The Application of Machine Learning Algorithms to Predict HIV Testing in Repeated Adult Population–Based Surveys in South Africa: Protocol for a Multiwave Cross-Sectional Analysis
Source: JMIR Res Protoc. 2025 Jan 27;14:e59916. doi: 10.2196/59916 (PMC11811654; doi:10.2196/59916)
Supplement: Multimedia Appendix 1 [file resprot_v14i1e59916_app1.docx]

STROBE Statement—checklist of items that should be included in reports of observational studies

|  | Item No. | Recommendation | Page  No. | Relevant text from manuscript |
| --- | --- | --- | --- | --- |
| **Title and abstract** | 1 | (*a*) Indicate the study’s design with a commonly used term in the title or the abstract | 1 | The Application of Machine Learning Algorithms to Predict HIV Testing in Repeated Adult Population-Based Surveys in South Africa: A Protocol for Multi-Wave Cross-Sectional Analysis |
|  |  | (*b*) Provide in the abstract an informative and balanced summary of what was done and what was found | 2 | This is a study protocol of a retrospective analysis of multiple cross-sectional survey data to identify the predictors of HIV testing among South African adults aged 18 years and above. An SML technique will be applied across the five cycles of the South African National HIV Prevalence, Incidence, Behavior, and Communication Survey (SABSSM) surveys. The Human Science Research Council (HSRC) conducted the SABSSM surveys in 2002, 2005, 2008, 2012 and 2017.  The findings of this study will identify consistent variables predicting HIV testing uptake among the South African adult population over the course of 20 years. Furthermore, the study will evaluate and compare the performance metrics of the four different ML algorithms, and the best model will be used to develop an HIV testing predictive model. |
| Introduction | | | |  |
| Background/rationale | 2 | Explain the scientific background and rationale for the investigation being reported | 3 - 5 | The human immunodeficiency virus-acquired immunodeficiency syndrome (HIV/AIDS) remains a public health threat, affecting 39 million people globally, of whom 25.6 million are within the Sub-Saharan African (SSA) region [1]. More than half of the global HIV prevalence is concentrated in East and Southern Africa, according to the World Health Organization (WHO) and the Joint United Nations Program on HIV/AIDS (UNAIDS) [1,2]. Human immunodeficiency virus testing is a crucial component of the HIV prevention and care continuum [3] and a pivotal step in realizing the UNAIDS's goal of ending AIDS by 2030 [4]. South Africa has made significant progress towards the UNAIDS 2030 target, ensuring 90% of people living with HIV become aware of their status by the end of 2022 [8,9]. However, the country remains behind schedule as the 90% attainment was set for 2020. Furthermore, there still exist numerous barriers to the uptake of HIV testing [10]. Although a significant amount of survey data is available on the factors associated with HIV testing in South Africa, there has been limited research on utilizing ML approaches to analyze and forecast these factors. Thus, it is against this backdrop that our study is pioneering in using four SML algorithms (logistic regression, random forest, SVM, and decision trees) across all five cycles of the SABSSM surveys to provide new insights into the determinants of HIV-testing behavior in South Africa. |
| Objectives | 3 | State specific objectives, including any prespecified hypotheses | 5 | The primary aim of this study is to identify consistent predictors of HIV testing and to compare the performance of the four SML models using repeated adult population-based surveys in South Africa in order to develop an evidence-based predictive model to enhance HIV testing. |
| Methods | | | |  |
| Study design | 4 | Present key elements of study design early in the paper | 5 | A retrospective analysis of data from multiple cross-sectional surveys will be used to predict factors associated with HIV testing across the five cycles of the SABSSM survey using SML algorithms. |
| Setting | 5 | Describe the setting, locations, and relevant dates, including periods of recruitment, exposure, follow-up, and data collection | 5-6 | This secondary data analysis will make use of the five cycles of the SABSSM surveys conducted by the South African Human Sciences Research Council (HSRC). The SABSSM surveys are a series of nationally representative surveys conducted periodically in South Africa to assess the prevalence, incidence, behaviors, and communication related to HIV/AIDS [10]. The first SABSSM survey was conducted in 2002 with 9,963 individuals interviewed, followed by SABSSM 2005 (*n*=23,275), SABSSM 2008 (*n*=20,826), SABSSM 2012 (*n*=38,431), and SABSSM 2017 (*n*=36,609) being the fifth survey [31-35]. All five cycles of the SABSSM datasets were made available to the research team on 20 August 2024 after filling and submitting the online request form via the HSRC website [33]. |
| Participants | 6 | (*a*) *Cohort study*—Give the eligibility criteria, and the sources and methods of selection of participants. Describe methods of follow-up  *Case-control study*—Give the eligibility criteria, and the sources and methods of case ascertainment and control selection. Give the rationale for the choice of cases and controls  *Cross-sectional study*—Give the eligibility criteria, and the sources and methods of selection of participants | 6 | The SABSSM dataset is a combination of children, adolescents, and adults’ data. This analysis will only include males and females aged 18 years and above from the SABSSM survey. Data outside the defined age bracket from the SABSSM survey data will be excluded from this analysis. |
|  |  | (*b*) *Cohort study*—For matched studies, give matching criteria and number of exposed and unexposed  *Case-control study*—For matched studies, give matching criteria and the number of controls per case | N/A |  |
| Variables | 7 | Clearly define all outcomes, exposures, predictors, potential confounders, and effect modifiers. Give diagnostic criteria, if applicable | 6-7 | The outcome variable for this study will be HIV testing status. The exposure variables are sociodemographic (age, gender, ethnicity/race, education level, marital status, employment, rural and urban residence), socioeconomic (household income, socioeconomic status, access and utilization of healthcare facilities), sexual behaviors (history of sexually transmitted infections, knowledge of HIV transmission, number of sexual partners, condom use), HIV knowledge and awareness (knowledge towards HIV prevention, testing and treatment services, knowledge of HIV status), and perceptions and attitudes (attitudes towards HIV testing, perceived risk of infection, stigma and discrimination related to HIV). |
| Data sources/ measurement | 8* | For each variable of interest, give sources of data and details of methods of assessment (measurement). Describe comparability of assessment methods if there is more than one group | 6-11 | This secondary data analysis will make use of the five cycles of the SABSSM surveys conducted by the South African HSRC in 2002, 2005, 2008, 2012, and 2017. The main outcome variable for this study is “HIV testing,” and the explanatory variables are sociodemographic, sociocultural, socioeconomic, behavioral, and individual factors. A machine learning technique will be used, and the results will be presented using frequencies, percentages, p values, odds ratios, accuracy, etc. |
| Bias | 9 | Describe any efforts to address potential sources of bias | 11-12 | Since the proposed study will analyze secondary data from multiple cross-sectional data, it is pertinent to assess and mitigate potential bias to ensure the validity and reliability of the results. The Joanna Briggs Institute (JBI) Critical Appraisal Checklist [this can be found in the Multimedia Appendix [JBI Critical Appraisal Checklist]. will be used to mitigate the risk of bias in this study. |
| Study size | 10 | Explain how the study size was arrived at | 6 | The final sample size for this study will be determined after cleaning and removing outliers from the datasets. |

Continued on next page

| Quantitative variables | 11 | Explain how quantitative variables were handled in the analyses. If applicable, describe which groupings were chosen and why | 9, 11-12 | Variables (features) with p<0.05 will be considered important predictors and will be retained in the final model for predicting HIV testing status. Potential confounders and redundant variables will also be addressed during the feature selection, as detailed in the risk for bias assessment section. |
| --- | --- | --- | --- | --- |
| Statistical methods | 12 | (*a*) Describe all statistical methods, including those used to control for confounding | 9-11 | The statistical analysis for this study includes Chi-squared, one-hot encoding, k-fold cross-validation, logistic regression, support vector machines, random forest, and decision trees. |
|  |  | (*b*) Describe any methods used to examine subgroups and interactions |  | N/A |
|  |  | (*c*) Explain how missing data were addressed | 9 | For this analysis, the research team will first clean and process the available data to handle outliers and missing values and identify relevant dependent and independent variables in the five cycles of the SABSSM survey to be included in the predictive modeling. This will be followed by feature selection to eliminate redundant and irrelevant variables. |
|  |  | (*d*) *Cohort study*—If applicable, explain how loss to follow-up was addressed  *Case-control study*—If applicable, explain how matching of cases and controls was addressed  *Cross-sectional study*—If applicable, describe analytical methods taking account of sampling strategy | 5 | A retrospective analysis of data from multiple cross-sectional surveys will be used to predict factors associated with HIV testing across the five cycles of the SABSSM survey using SML algorithms. The analysis will involve four SML algorithms in developing an HIV testing predictive model for the South African adult population. |
|  |  | (*e*) Describe any sensitivity analyses | 10 | Upon completing the model development, the performance of the four SML models will be assessed on the testing sample using standardized performance evaluation metrics, including accuracy, precision, recall, f-1 score, AUC-ROC, and confusion matrix. |
| Results | | | | |
| Participants | 13* | (a) Report numbers of individuals at each stage of study—eg numbers potentially eligible, examined for eligibility, confirmed eligible, included in the study, completing follow-up, and analysed | 13 | Since this is a protocol, the sample will be determined after cleaning the data for analysis.  The results of this study will be available by 31 January 2025, and each step of the analysis will be visualized and presented in tables and graphs. The anticipated date of publication for the proposed study is June 2025. |
|  |  | (b) Give reasons for non-participation at each stage |  | N/A |
|  |  | (c) Consider use of a flow diagram |  | N/A |
| Descriptive data | 14* | (a) Give characteristics of study participants (eg demographic, clinical, social) and information on exposures and potential confounders |  | N/A |
|  |  | (b) Indicate number of participants with missing data for each variable of interest |  | N/A |
|  |  | (c) *Cohort study*—Summarise follow-up time (eg, average and total amount) |  | N/A |
| Outcome data | 15* | *Cohort study*—Report numbers of outcome events or summary measures over time |  | N/A |
|  |  | *Case-control study—*Report numbers in each exposure category, or summary measures of exposure |  | N/A |
|  |  | *Cross-sectional study—*Report numbers of outcome events or summary measures |  | N/A |
| Main results | 16 | (*a*) Give unadjusted estimates and, if applicable, confounder-adjusted estimates and their precision (eg, 95% confidence interval). Make clear which confounders were adjusted for and why they were included |  | N/A |
|  |  | (*b*) Report category boundaries when continuous variables were categorized |  | N/A |
|  |  | (*c*) If relevant, consider translating estimates of relative risk into absolute risk for a meaningful time period |  | N/A |

Continued on next page

| Other analyses | 17 | Report other analyses done—eg analyses of subgroups and interactions, and sensitivity analyses |  | N/A |
| --- | --- | --- | --- | --- |
| Discussion | | | | |
| Key results | 18 | Summarise key results with reference to study objectives | 14 | The proposed study seeks to identify intricate patterns and relationships among various socio-demographic, behavioral, and contextual variables that contribute to individuals' decisions to undergo HIV testing in South Africa. Since the first SABSSM survey in 2002, varying rates of HIV testing among South Africans have been reported by the HSRC, showing increased trends in each cycle until 2017 (Table 2). By applying SML learning techniques across the five cycles of the SABSSM survey datasets, the findings of this study will ascertain consistent variables predicting HIV testing uptake among the South African adult population over the 20-year period. Furthermore, the study will evaluate and compare the performance metrics of the four different SML algorithms, and the best model will be used to develop an evidence-based predictive model to enhance HIV testing among the South African adult population. |
| Limitations | 19 | Discuss limitations of the study, taking into account sources of potential bias or imprecision. Discuss both direction and magnitude of any potential bias | 15 | To the best of our knowledge, this will be the first study to conduct HIV testing predictive modeling using all five cycles of the SABSSM surveys by applying SML algorithms. The large volume of data will be crucial for the generalizability of the developed models as well as the major findings. It is also important to mention that the research involves a multidisciplinary team of experts across public health, epidemiology, biostatistics, and data science. The well-equipped team will devise a comprehensive methodological approach to developing an adaptable HIV testing predictive model.  However, this study is expected to face some limitations as the SABSSM surveys may contain missing values; proper imputation techniques will be used to address this issue. Since some ML models are not well developed, causing spatial and temporal variations, this could also be a limitation in our proposed study. Furthermore, because the analysis focuses on secondary datasets gathered using cross-sectional designs, the researchers are aware of potential limitations such as biases and lack of control over the variables included. The JBI Critical Appraisal Checklist will be used to mitigate the risk of bias in this study. |
| Interpretation | 20 | Give a cautious overall interpretation of results considering objectives, limitations, multiplicity of analyses, results from similar studies, and other relevant evidence |  | This will be available after the analysis. |
| Generalisability | 21 | Discuss the generalisability (external validity) of the study results | 15 | The large volume of data will be crucial for the generalizability of the developed models as well as the major findings. |
| Other information | |  | | |
| Funding | 22 | Give the source of funding and the role of the funders for the present study and, if applicable, for the original study on which the present article is based | 16 | *“The research outlined here was made possible through funding from SAMRC under its Division of Research Capacity Development, supported by the South African National Treasury (Project Code number: 57035, SAMRC File ref no: HDID8528/KR/202). This work was conducted under the auspices of the SAMRC/UJ PACER Extramural Unit. The content of this paper remains the sole responsibility of the authors and does not necessarily represent the official views of SAMRC or UJ. Additionally, this paper forms an integral part of Mr. Musa Jaiteh's PhD study and is generously funded by the GES 4.0 Scholarship at UJ.”* |

*Give information separately for cases and controls in case-control studies and, if applicable, for exposed and unexposed groups in cohort and cross-sectional studies.

**Note:** An Explanation and Elaboration article discusses each checklist item and gives methodological background and published examples of transparent reporting. The STROBE checklist is best used in conjunction with this article (freely available on the Web sites of PLoS Medicine at http://www.plosmedicine.org/, Annals of Internal Medicine at http://www.annals.org/, and Epidemiology at http://www.epidem.com/). Information on the STROBE Initiative is available at www.strobe-statement.org.
