# Supplementary material for: The Application of Machine Learning Algorithms to Predict HIV Testing in Repeated Adult Population–Based Surveys in South Africa: Protocol for a Multiwave Cross-Sectional Analysis
Source: JMIR Res Protoc. 2025 Jan 27;14:e59916. doi: 10.2196/59916 (PMC11811654; doi:10.2196/59916)
Supplement: Multimedia Appendix 5 [file resprot_v14i1e59916_app5.docx]

Tables S1-S7 contain the outcome and exposure variables that are present in the 2008 SABSSM surveys

**Table S1. Variable Definition: Main outcome Variable (HIV Testing)**

| **Variable Name** | **Variables** | **Variable Descriptions** | **Variable Recode** |
| --- | --- | --- | --- |
|  |  |  |  |
| HIV Testing | Ever had an HIV Test | 1) Yes  2) No  3) No response | 1) Yes  2) No |

**Table S2. Variable Definition: Exposure Variables (Socio-demographic)**

| **Variable Name** | **Variables** | **Variable Descriptions** | **Variable Recode** |
| --- | --- | --- | --- |
|  |  |  |  |
| Age | Respondent’s Age in years | Integer | 1) <20  2) 20-24  3) 25-29  4) 30-34  5) 35-39  6) 40-44  7) 45-49  8) ≥50 |
| Province | Province | 1) Western Cape  2) Eastern Cape  3) Northern Cape  4) Free State  5) KwaZulu-Natal  6) North-West  7) Gauteng  8) Mpumalanga  9) Limpopo | 1) Western Cape  2) Eastern Cape  3) Northern Cape  4) Free State  5) KwaZulu-Natal  6) North-West  7) Gauteng  8) Mpumalanga  9) Limpopo |
| geotype | Geographical location | 1) Urban formal  2) Urban informal  2) Rural formal  3) Rural informal | 1) Urban  2) Rural |
| Race | Race | 1) African  2) White  3) Coloured  4) Indian/ Asian  5) Other | 1) African  2) Coloured  3) Indian  4) White |
| Sex | Sex of the respondent | 1) Male  2) Female | 1) Male  2) Female |
| Highest level of education | Respondent’s highest level of education obtained | 1. No schooling  2. Up to Std 1/Gr 3 / ABET 1  3. Std 2 - Std 3/ Gr 4 - Gr 5 / ABET 2  4. Std 4 - Std 5/ Gr 6 - Gr 7 / ABET 3  5. Std 6 - Std 7/ Gr 8 - Gr 9 / ABET 4  6. Std 8/ Gr 10 / NTC 1  7. Std 9/ Gr 11 / NTC 2  8. Std 10/ Gr 12/ Matric/ NTC 3  9. Certificate or Diploma with Gr 12  10. Bachelors Degree  11. Post-graduate degree (HDE/Hons/Masters/PhD) | 1. No schooling  2. Primary  3. Secondary  4. Tertiary |
| marital status | Current marital status | 1) Married - Civil (magistrate)  2) Married - Traditional (lobola or dowry)  3) Married – Religious  4) Married - Civil and traditional (lobola or dowry)  5) Married - Civil and religious  6) Single  7) Divorced / separated  8) Living together / not married  9) Widower / Widow  10) Civil Unions  11) Other (specify): | 1) Married  2) Single  3) Divorced/ Separated  4) Widower/ widow |

**Table S3. Variable Definition: Exposure Variables (Sociocultural)**

| **Variable Name** | **Variables** | **Variable Descriptions** | **Variable Recode** |
| --- | --- | --- | --- |
| Age at first marriage | How old was the respondent when married for the first time | Integer | 1) <18  2) 18 -23  3) 24 -29  4) 30 -35  5) > 35 |
| Male circumcision | Whether the male respondent is circumcised | 1. yes  2. No | 1. yes  2. No |
| Type of circumcision | Repontdent’s type of circumcision | 1. Partial  2. Full/ Complete (foreskin total removed)  3. Don’t know | 1. Partial  2. Full/ Complete (foreskin total removed)  3. Don’t know |

**Table S4. Variable Definition: Exposure Variables (Socioeconomic)**

| **Variable Name** | **Variables** | **Variable Descriptions** | **Variable Recode** |
| --- | --- | --- | --- |
| Employment status | Employment status | 1. Housewife, homemaker, not looking for work  2. Housewife, homemaker, looking for work  3. Unemployed, looking for work  4. Unemployed, not looking for work  5. Work in informal sector, not looking for permanent work  6. Work in informal sector, not looking for permanent work  7. Sick/disabled and unable to work  8. Student/pupil/learner  9. Self-employed - full-time (40 hours or more per week)  10. Self-employed - part-time (less than 40 hours per week)  11. Employed part-time (if none of the above) (less than 40 hours per week)  12. Employed full-time (40 hours or more per week)  13. Other | 1. Unemployed  2. Sick/ disabled and unable to work  3. Student/ pupil/ learner  4. Employed/ Self-employed  4. Other |
| Gross annual income | Respondents' gross annual income | 1. No income  2. Less than R6 000 per year (Less than R500 per month)  3. R6 001 – R12 000 per year (R501 to R1 000 per month)  4. R12 001 – R24 000 per year (R1 001 to R2 000 per month)  5. R24 001 - R48 000 per year (R2 001 to R4 000 per month)  6. R48 001 – R96 000 per year (R4 001 to R8 000 per month)  7. R96 001 – R192 000 per year (R8 001 to R16 000 per month)  8. R192 001 – R384 000 per year (R16 000 to R32 000 per month)  9. More than R384 001 per year (More than R32 000 per month) | 1. No income  2. Less than R6 000 per year (Less than R500 per month)  3. R6 001 – R12 000 per year (R501 to R1 000 per month)  4. R12 001 – R24 000 per year (R1 001 to R2 000 per month)  5. R24 001 - R48 000 per year (R2 001 to R4 000 per month)  6. R48 001 – R96 000 per year (R4 001 to R8 000 per month)  7. R96 001 – R192 000 per year (R8 001 to R16 000 per month)  8. R192 001 – R384 000 per year (R16 000 to R32 000 per month)  9. More than R384 001 per year (More than R32 000 per month) |
| Gross monthly income | Respondents' gross monthly income | Numeric | 1. < R 5000  2. R 5000 – R 9 000  3. R 10 000 – R 14 000  4. R 15 000 -R 19 000  5. R 20 000 – R 24 000  6. R 25 000 – R 29 000  7. R 30 000 or more |
| Partner’s employment status | What is the respondent partner's employment status | 1. Employed  2. Unemployed  3. Student  98. Don’t know | 1. Employed  2. Unemployed  3. Student  98. Don’t know |

**Table S5. Variable Definition: Exposure Variables (Sexual History, Sexual Behaviour & Lifestyle)**

| **Variable Name** | **Variables** | **Variable Descriptions** | **Variable Recode** |
| --- | --- | --- | --- |
| Ever had a sexual intercourse | Whether the respondent ever had sexual intercourse | 1. Yes  2.No  3. No Response | 1. Yes  2.No |
| Age at first sex | Age of respondent at first sexual intercourse | Integers  1. Cannot remember age | 1. < 14 year  2. 15 -19 years  3. 20 -24  4. 25 – 30  5. > 30  6. Cannot remember age |
| Number of sexual partners in a lifetime | Number of people the respondent had sexual intercourse with in a lifetime | Integers | 1. 1 person  2. 2 -5  3. > 5 |
| Condom use at first sex | Whether respondent used condom at first sex | 1. Yes  2. No  3. Canno remember | 1. Yes  2. No  3. Canno remember |
| Had sex during the last 12 months | Whether respondent Had sex during the last 12 months | 1. Yes  2. No  3. No Response | 1. Yes  2. No  3. No Response |
| Number of sexual partners during the past 12 months | Number of sexual partners the respondent had during the past 12 months | Integers | 1. 1 person  2. 2 -5  3. > 5 |
| Number of male sexual partners during the past 12 months | Number of male sexual partners the respondent had during the past 12 months | Integers | 1. 1 person  2. 2 -5  3. > 5 |
| Number of female sexual partners during the past 12 months | Number of female sexual partners the respondent had during the past 12 months | Integers | 1. 1 person  2. 2 -5  3. > 5 |
| Received money or gifts or favours in exchange for sex | Whether the respondent received money or gifts or favours in exchange for sex | 1. Yes  2. No  3. Don’t know | 1. Yes  2. No  3. Don’t know |
| Two or more sexual partners at the moment | Whether the respondent has two or more sexual partners at the moment | 1. Yes  2. No  3. No response | 1. Yes  2. No  3. No response |
| Sexual partners in the last 3 months | The number of sexual partners the respondent has had in the last 3 months | Integers | 1. 1 person  2. 2 -5  3. > 5 |
| Condom use by any of the partners in the last 3 months | Whether any of the respondent’s sexual partners in the last 3 months used a condom. | 1. Yes  2. No | 1. Yes  2. No |
| Condom break/leak/slip | Whether the respondent experienced condom leak/break/slip off during sex or pulling out | 1. Yes  2. No  3. Don’t know | 1. Yes  2. No  3. Don’t know |
| Frequency of condom use | How often a respondent uses a condom with most recent partner | 1. Every time  2. Almost every time  3. Sometimes  4. Never | 1. Every time  2. Almost every time  3. Sometimes  4. Never |
| Condom use at last sex | Did you use a condom at last sex? Most recent person | 1. Yes  2. No | 1. Yes  2. No |
| Condom use decision | Who suggested using a condom? Second most recent person | 1. Yourself  2. Your Partner  3. Mutual agreement | 1. Yourself  2. Your Partner  3. Mutual agreement |
| Reasons for condom use | If you used a condom, what were your reasons for doing so? Most recent person | 1. Concern about HIV infection  2. People are urged to use condoms  3. Want to prevent STIs  4. Want to prevent pregnancy  5. Other | 1. Concern about HIV infection  2. People are urged to use condoms  3. Want to prevent STIs  4. Want to prevent pregnancy  5. Other |
| Reasons for not using a condom | If you did not use a condom, what were your reasons for not doing so? Most recent person | 1. Did not have a condom  2. Partner objected  3. Used other contraceptive  4. Don't like them  5. Didn't think it was necessary  6. I am married  7. I am faithful /trust them  8. I was drunk/high  8. Other | 1. Did not have a condom  2. Partner objected  3. Used other contraceptive  4. Don't like them  5. Didn't think it was necessary  6. I am married  7. I am faithful /trust them  8. I was drunk/high  8. Other |
| Drink alcohol at last sex | The last time you had sex with your partner, did you drink alcohol before sex - Most recent person | 1. Yes  2. No  3. Can't remember | 1. Yes  2. No  3. Can't remember |
| Condom access | Is it easy to get a condom if you need one? (Male and/female condoms) | 1. Yes  2. No  3. No response | 1. Yes  2. No |
| A place where the condom is obtained | Where do you normally obtain condoms? | 1. Government clinic or hospital  2. Private clinic or hospital  3. Pharmacy/chemist  4. Shop/supermarket/cafe  5. Garage/filling station  6. Spaza shop  7. Shebeen / tavern / hotel  8. Other | 1. Government clinic or hospital  2. Private clinic or hospital  3. Pharmacy/chemist  4. Shop/supermarket / cafe  5. Garage/filling station  6. Spaza shop  7. Shebeen / tavern / hotel  8. Other |
| Paid for a condom used | Whether the respondent or the partner paid for the last condom they used or got it for free | 1. Paid for  2. Free  3. Not sure/ don’t know | 1. Paid for  2. Free  3. Not sure/ don’t know |
| Drink alcohol | Ever had a drink containing alcohol | 1. Yes  2. No | 1. Yes  2. No |
| Frequency of alcohol intake | How often do you have a drink containing alcohol in the past 12 months? | 1. Not in the past 12 months  2. Once a month or less  3. 2-4 times a month  4. 2-3 times a week  5. 4 or more times a week | 1. Not in the past 12 months  2. Once a month or less  3. 2-4 times a month  4. 2-3 times a week  5. 4 or more times a week |
| Number of alcohol drinks | How many drinks containing alcohol do you have on a typical day when you are drinking? | 1. 1 or 2  2. 3 or 4  3. 5 or 6  4. 7 to 9  5. 10 or more | 1. 1 or 2  2. 3 or 4  3. 5 or 6  4. 7 to 9  5. 10 or more |
| Alcohol-related violence | Have you or someone else been injured as a result of your drinking? | 1. No  2. Yes, but not in the las 12 months  3. Yes, during the last 12 months | 1. No  2. Yes, but not in the las 12 months  3. Yes, during the last 12 months |
| Smoke cannabis | How often during the past 12 months did you feel guilt or remorse after drinking? | 1. Never  2. less than a month  3. Monthly  4. Weekly  5. Daily or almost daily | 1. Never  2. less than a month  3. Monthly  4. Weekly  5. Daily or almost daily |
| Share injections | Have you ever shared needle injections? | 1. No, never  2. Yes, in the past 3 months  3. Yes, but not in the past 3 months | 1. No, never  2. Yes, in the past 3 months  3. Yes, but not in the past 3 months |
| Still sexually active with partner | Are you still sexually active with your partner? Most recent person | 1. Yes  2. No | 1. Yes  2. No |

**Table S6. Variable Definition: Exposure Variables (Health status/ Pre-existing medical conditions/ disabilities/ Stigma/ Violence)**

| **Variable Name** | **Variables** | **Variable Descriptions** | **Variable Recode** |
| --- | --- | --- | --- |
| Disability status | Whether the respondent has a disability | 1. Yes  2. No  3. Unsure | 1. Yes  2. No  3. Don’t know |
| Type of disability | What is the disability? | 1. Physical (spinal injury, loss of a limb, etc.)  2. Sight  3. Partial hearing  4. communication/ speech  5. Mental or psychiatric illness | 1. Physical (spinal injury, loss of a limb, etc.)  2. Sight  3. Partial hearing  4. communication/ speech  5. Mental or illness |
| PAP smear test | Have you ever had a test for a PAP smear? (By PAP smear test, I mean did a doctor or nurse use a swab or stick to wipe from inside your vagina, take a sample and send it to the laboratory). | 1. Yes  2. No | 1. Yes  2. No |
| Current pregnancy status | Are you pregnant now? | 1. Yes  2. No  3. Unsure | 1. Yes  2. No  3. Unsure |
| Visist clinic during current pregnancy | Have you visited an antenatal clinic during this pregnancy? | 1. Yes  2. No | 1. Yes  2. No |
| Offered HIV test | During this current pregnancy, was an HIV test offered to you during any of your antenatal care clinic visits? | 1. Yes  2. No  3. Do not know | 1. Yes  2. No  3. Do not know |
| Tested during current pregnancy | During this current pregnancy, have you been tested for HIV during any of your antenatal care clinic visits? | 1. Yes  2. No | 1. Yes  2. No |
| General wellbeing | In general, would you say that your health is excellent, good, fair or poor? | 1. Excellent  2. Good  3. Fair  4. Poor | 1. Excellent  2. Good  3. Fair  4. Poor |
| Hypertension | Hypertension/high blood pressure - Diagnosed with illness | 1. Yes  2. No  3. Refused to answer | 1. Yes  2. No  3. Refused to answer |
| Sexual violence by partner | Did your partner ever - Physically force you to have sexual intercourse with him/her | 1. Yes  2. No | 1. Yes  2. No |

**Table S7. Variable Definition: Exposure Variables (Knowledge, Awareness, and Perception of HIV/AIDS)**

| **Variable Code/ Name** | **Variables** | **Variable Descriptions** | **Variable Recode** |
| --- | --- | --- | --- |
| Knowledge - HIV prevention | Whether respondent knows ways HIV can be prevented | 1. It can’t be prevented  2. Using condoms  3. Sticking to one sex partner  4. Being faithful to one sex partner who is also faithful to you  5. Reducing number of sex partners  6. Abstaining from sex  7. Avoiding contact with blood  8. Using drugs to prevent HIV transmission from mother to child  9. Medical male circumcision (as an HIV prevention method)  10. Microbicides (gel/ring inserted into the vagina to prevent HIV infection)  11. PrEP (taking ARVs to prevent HIV infection)  12. Other  13. I don’t know | 1. It can’t be prevented  2. Using condoms  3. Sticking to one sex partner  4. Being faithful to one sex partner who is also faithful to you  5. Reducing number of sex partners  6. Abstaining from sex  7. Avoiding contact with blood  8. Using drugs to prevent HIV transmission from mother to child  9. Medical male circumcision (as an HIV prevention method)  10. Microbicides (gel/ring inserted into the vagina to prevent HIV infection)  11. PrEP (taking ARVs to prevent HIV infection)  12. Other  13. I don’t know |
| Knowledge - AIDS | Can AIDS be cured? | 1. Yes  2. No  3. I don’t know | 1. Yes  2. No  3. I don’t know |
| Knowledge – HIV risk | Can a person reduce the risk of HIV by having fewer sexual partners? | 1. Yes  2. No  3. I don’t know | 1. Yes  2. No  3. I don’t know |
| Knowledge – HIV acquisition | Can a healthy-looking person have HIV? | 1. Yes  2. No  3. I don’t know | 1. Yes  2. No  3. I don’t know |
| Knowledge - PMTCT | Can HIV be transmitted from a mother to her unborn baby? | 1. Yes  2. No  3. I don’t know | 1. Yes  2. No  3. I don’t know |
| Knowledge – HIV transmission | Can the risk of HIV transmission be reduced by having sex with only one uninfected partner who has no other partners? | 1. Yes  2. No  3. I don’t know | 1. Yes  2. No  3. I don’t know |
| Knowledge – HIV transmission | Can a person get HIV by sharing food with someone who is infected? | 1. Yes  2. No  3. I don’t know | 1. Yes  2. No  3. I don’t know |
| Knowledge – HIV prevention by condom use | Can a person reduce the risk of getting HIV by using a condom every time he/she has sex? | 1. Yes  2. No  3. I don’t know | 1. Yes  2. No  3. I don’t know |
| Knowledge – HIV prevention by male circumcision | Can medical male circumcision reduce the risk of HIV infection in males? | 1. Yes  2. No  3. I don’t know | 1. Yes  2. No  3. I don’t know |
| Awareness of HIV treatment | Have you heard about drug treatments HIV positive pregnant women can take to reduce risk of infecting the baby? | 1) Yes  2) No | 1) Yes  2) No |
| Awareness of HIV treatment | Have you heard about drug treatments that can help reduce the risk of HIV infection if a person has been raped? | 1) Yes  2) No | 1) Yes  2) No |
| Know a place to get an HIV test | Whether respondents know a place nearby where they can get an HIV test | 1) Yes  2) No | 1) Yes  2) No |
| Comfortable in talking about HIV/AIDS | Are you comfortable talking to at least one member of your family about HIV/AIDS? | 1) Yes  2) No  3) Not sure | 1) Yes  2) No  3) Not sure |
| Perception of politician influence in HIV control | Political leaders are committed to controlling HIV/AIDS in South Africa | 1. Agree  2. Disagree  3. Do not know | 1. Agree  2. Disagree  3. Do not know |
| Perception of Government Support of HIV | The government supports people and families living with HIV/AIDS | 1. Agree  2. Disagree  3. Do not know | 1. Agree  2. Disagree  3. Do not know |
| Perceived risk of becoming infected with HIV | On a scale of 1 to 4 (with 1 being low and 4 being high), how would you rate yourself in terms of risk of becoming infected with HIV? | 4. You are definitely going to get infected with HIV  3. You are probably going to get infected  2. You probably won’t get infected  1. You definitely will not get infected with HIV  9. Already HIV positive | 4. You are definitely going to get infected with HIV  3. You are probably going to get infected  2. You probably won’t get infected  1. You definitely will not get infected with HIV  9. Already HIV positive |
