# Supplementary material for: The Application of Machine Learning Algorithms to Predict HIV Testing in Repeated Adult Population–Based Surveys in South Africa: Protocol for a Multiwave Cross-Sectional Analysis
Source: JMIR Res Protoc. 2025 Jan 27;14:e59916. doi: 10.2196/59916 (PMC11811654; doi:10.2196/59916)
Supplement: Multimedia Appendix 7 [file resprot_v14i1e59916_app7.docx]

Tables S1-S7 contain the outcome and exposure variables that are present in the 2017 SABSSM surveys

**Table S1. Variable Definition: Main outcome Variable (HIV Testing)**

| **Variable Name** | **Variables** | **Variable Descriptions** | **Variable Recode** |
| --- | --- | --- | --- |
|  |  |  |  |
| HIV Testing | Ever had an HIV Test | 1) Yes  2) No  3) No response | 1) Yes  2) No |

**Table S2. Variable Definition: Exposure Variables (Socio-demographic)**

| **Variable Name** | **Variables** | **Variable Descriptions** | **Variable Recode** |
| --- | --- | --- | --- |
|  |  |  |  |
| Age | Respondent’s Age in years | Integer | 1) <20  2) 20-24  3) 25-29  4) 30-34  5) 35-39  6) 40-44  7) 45-49  8) ≥50 |
| Province | Province | 1) Western Cape  2) Eastern Cape  3) Northern Cape  4) Free State  5) KwaZulu-Natal  6) North-West  7) Gauteng  8) Mpumalanga  9) Limpopo | 1) Western Cape  2) Eastern Cape  3) Northern Cape  4) Free State  5) KwaZulu-Natal  6) North-West  7) Gauteng  8) Mpumalanga  9) Limpopo |
| geotype | Geographical location | 1) Urban  2) Rural informal (tribal areas)  3) Rural (farms) | 1) Urban  2) Rural |
| Race | Race | 1) African  2) Coloured  3) Indian  4) White | 1) African  2) Coloured  3) Indian  4) White |
| Sex | Sex of the respondent | 1) Male  2) Female | 1) Male  2) Female |
| Gender | Gender of the respondent | 1) Male  2) Female  3) Transgender  4) Intersex | 1) Male  2) Female  3) Transgender  4) Intersex |
| Ever attended school | Whether the respondent ever attended school | 1. Yes  2. No | 1. Yes  2. No |
| Currently attending school/ post-school | Whether the respondent is currently attending school/ post-school | 1. Yes  2. No | 1. Yes  2. No |
| marital status | Marital status | 1) Married  2) Never Married  3) Divorced/ Separated  4) Widower/ widow | 1) Married  2) Never Married  3) Divorced/ Separated  4) Widower/ widow |

**Table S3. Variable Definition: Exposure Variables (Sociocultural)**

| **Variable Name** | **Variables** | **Variable Descriptions** | **Variable Recode** |
| --- | --- | --- | --- |
| Age at first marriage | How old was the respondent when married for the first time | Integer | 1) <18  2) 18 -23  3) 24 -29  4) 30 -35  5) > 35 |
| Living arrangement | Current living agreement | 1. Living with husband/wife  2. Living on own or other arrangement but not living with husband/wife  3. Living together with boyfriend/girlfriend/civil union (same sex) partner /partner /another partner  4. Single/ divorced/ widowed – in a steady relationship but not living together  5. Single; not in a steady relationship | 1. Living with husband/wife  2. Living on own or other arrangement but not living with husband/wife  3. Living together with boyfriend/girlfriend/civil union (same sex) partner /partner /another partner  4. Single/ divorced/ widowed – in a steady relationship but not living together  5. Single; not in a steady relationship |
| In a polygamous union | Whether the respondent is in a polygamous union | 1. Yes  2. No | 1. Yes  2. No |
| Number of wives | Number of wives husband has/ number of co-wives | Integer | 1. 1 wife  2. 2 wives  3. 3 wives  4. > 3 wives |
| Male circumcision | Whether male respondent is circumcised | 1. yes  2. No | 1. yes  2. No |
| Type of circumcision | Repontdent’s type of circumcision | 1. Partial (where some of the foreskin still remain)  2. Full/ Complete (foreskin total removed)  3. Don’t know | 1. Partial (where some of the foreskin still remain)  2. Full/ Complete (foreskin total removed)  3. Don’t know |

**Table S4. Variable Definition: Exposure Variables (Socioeconomic)**

| **Variable Name** | **Variables** | **Variable Descriptions** | **Variable Recode** |
| --- | --- | --- | --- |
| Highest level of education | Respondent’s highest level of education obtained | 1. Pre-school/ Gr R  2. Garde 1/ Sub a/ Class 1  3. Grade 2/ Sub b/ Class 2  4. Grade 3/ Standard 1/ Abet 1  5. Grade 4/ Standard 2/ Abet 2  6. Grade 5/ Standard 3/ Abet 2  7. Grade 6/ Standard 4/ Abet 3  8. Grade 7/ Standard 5/ Abet 3  9. Grade 8/ Standard 6/ Abet 3  10. Grade 9/ Standard 7/ Abet 3  11. Grade 10/ Standard 8/ Ntc 1  12. Grade 11/ Standard 9/ Ntc 2  13. Grade 12/ Standard 10/ Ntc 3  14. 15. Further studies incomplete  15. Diploma/ undergraduate degree/ other post-school completed  16. Further degree completed  17. Don’t know | 1. Pre-school/ Gr R  2. Primary  3. Secondary  4. Tertiary |
| Employment status | Employment status | 1. Unemployed  2. Sick/ disabled and unable to work  3. Student/ pupil/ learner  4. Employed/ Self-employed  4. Other | 1. Unemployed  2. Sick/ disabled and unable to work  3. Student/ pupil/ learner  4. Employed/ Self-employed  4. Other |
| Received income in the previous month | Whether respondent received income from any source in the previous month | 1. Yes  2. No | 1. Yes  2. No |
| Main source of income in the previous month | Respondent’s main source of income in the previous month | 1. Salary/earnings  2. Contributions by family members or relatives  3. Government pensions/grants (e.g., old age pension, child support grant, disability grant)  4. Grants/donations by private welfare organizations  5. Other sources (Specify?......)  6. No income | 1. Salary/earnings  2. Contributions by family members or relatives  3. Government pensions/grants (e.g., old age pension, child support grant, disability grant)  4. Grants/donations by private welfare organizations  5. Other sources (Specify?......)  6. No income |
| Gross monthly income | Respondents' gross monthly income | Numeric | 1. < R 5000  2. R 5000 – R 9 000  3. R 10 000 – R 14 000  4. R 15 000 -R 19 000  5. R 20 000 – R 24 000  6. R 25 000 – R 29 000  7. R 30 000 or more |
| Partner’s employment status | What is the respondent partner's employment status | 1. Employed  2. Unemployed  3. Student  98. Don’t know | 1. Employed  2. Unemployed  3. Student  98. Don’t know |

**Table S5. Variable Definition: Exposure Variables (Sexual History, Sexual Behaviour & Lifestyle)**

| **Variable Name** | **Variables** | **Variable Descriptions** | **Variable Recode** |
| --- | --- | --- | --- |
| Ever had a sexual intercourse | Whether the respondent ever had sexual intercourse (vaginal or anal) | 1. Yes  2.No  3. No Response | 1. Yes  2.No |
| Age at first sex | Age of respondent at first sexual intercourse | Integers  1. Cannot remember age | 1. < 14 year  2. 15 -19 years  3. 20 -24  4. 25 – 30  5. > 30  6. Cannot remember age |
| Number of sexual partners in a lifetime | Number of people the respondent had sexual intercourse with in a lifetime | Integers | 1. 1 person  2. 2 -5  3. > 5 |
| Condom use at first sex | Whether respondent used condom at first sex | 1. Yes  2. No  3. Canno remember | 1. Yes  2. No  3. Canno remember |
| Had sex during the last 12 months | Whether respondent Had sex during the last 12 months | 1. Yes  2. No  3. No Response | 1. Yes  2. No  3. No Response |
| Number of sexual partners during the past 12 months | Number of sexual partners the respondent had during the past 12 months | Integers | 1. 1 person  2. 2 -5  3. > 5 |
| Number of male sexual partners during the past 12 months | Number of male sexual partners the respondent had during the past 12 months | Integers | 1. 1 person  2. 2 -5  3. > 5 |
| Number of female sexual partners during the past 12 months | Number of female sexual partners the respondent had during the past 12 months | Integers | 1. 1 person  2. 2 -5  3. > 5 |
| Received money or gifts or favours in exchange for sex | Whether the respondent received money or gifts or favours in exchange for sex | 1. Yes  2. No  3. Don’t know | 1. Yes  2. No  3. Don’t know |
| Two or more sexual partners at the moment | Whether the respondent has two or more sexual partners at the moment | 1. Yes  2. No  3. No response | 1. Yes  2. No  3. No response |
| Sexual partners in the last 3 months | The number of sexual partners the respondent has had in the last 3 months | Integers | 1. 1 person  2. 2 -5  3. > 5 |
| Condom use by any of the partners in the last 3 months | Whether any of the respondent’s sexual partners in the last 3 months used a condom. | 1. Yes  2. No | 1. Yes  2. No |
| Condom break/leak/slip | Whether the respondent experienced condom leak/break/slip off during sex or pulling out | 1. Yes  2. No  3. Don’t know | 1. Yes  2. No  3. Don’t know |
| Frequency of condom use | How often a respondent uses a condom with a partner | 1. Every time  2. Almost every time  3. Sometimes  4. Never | 1. Every time  2. Almost every time  3. Sometimes  4. Never |
| Condom use at last sex | Did you use a condom at last sex? Most recent person | 1. Yes  2. No | 1. Yes  2. No |
| Condom use decision | Who suggested using a condom? Second most recent person | 1. Yourself  2. Your Partner  3. Mutual agreement | 1. Yourself  2. Your Partner  3. Mutual agreement |
| Reasons for condom use | If you used a condom, what were your reasons for doing so? Most recent person | 1. Concern about HIV infection  2. People are urged to use condoms  3. Want to prevent STIs  4. Want to prevent pregnancy  5. I or partner on ARV  6. Other | 1. Concern about HIV infection  2. People are urged to use condoms  3. Want to prevent STIs  4. Want to prevent pregnancy  5. I or partner on ARV  6. Other |
| Reasons for not using a condom | If you did not use a condom, what were your reasons for not doing so? Most recent person | 1. Did not have a condom  2. Partner objected  3. Used other contraceptive  4. Don't like them  5. Didn't think it was necessary  6. I am married  7. I am faithful /trust them  8. I was drunk/high  8. Other | 1. Did not have a condom  2. Partner objected  3. Used other contraceptive  4. Don't like them  5. Didn't think it was necessary  6. I am married  7. I am faithful /trust them  8. I was drunk/high  8. Other |
| Drink alcohol at last sex | The last time you had sex with your partner, did you drink alcohol before sex - Most recent person | 1. Yes  2. No  3. Can't remember | 1. Yes  2. No  3. Can't remember |
| Condom access | Is it easy to get a condom if you need one? (Male and/female condoms) | 1. Yes  2. No  3. No response | 1. Yes  2. No |
| A place where the condom is obtained | Where do you normally obtain condoms? | 1. Government clinic or hospital  2. Private clinic or hospital  3. Pharmacy/chemist  4. Shop/supermarket/cafe  5. Garage/filling station  6. Spaza shop  7. Shebeen / tavern / hotel  8. Workplace  9. Other | 1. Government clinic or hospital  2. Private clinic or hospital  3. Pharmacy / chemist  4. Shop / supermarket / cafe  5. Garage / filling station  6. Spaza shop  7. Shebeen / tavern / hotel  8. Workplace  9. Other |
| Paid for a condom used | Whether the respondent or the partner paid for the last condom they used or got it for free | 1. Paid for  2. Free  3. Not sure/ don’t know | 1. Paid for  2. Free  3. Not sure/ don’t know |
| Drink alcohol | Ever had a drink containing alcohol | 1. Yes  2. No | 1. Yes  2. No |
| Frequency of alcohol intake | How often do you have a drink containing alcohol in the past 12 months? | 1. Not in the past 12 months  2. Once a month or less  3. 2-4 times a month  4. 2-3 times a week  5. 4 or more times a week | 1. Not in the past 12 months  2. Once a month or less  3. 2-4 times a month  4. 2-3 times a week  5. 4 or more times a week |
| Number of alcohol drinks | How many drinks containing alcohol do you have on a typical day when you are drinking? | 1. 1 or 2  2. 3 or 4  3. 5 or 6  4. 7 to 9  5. 10 or more | 1. 1 or 2  2. 3 or 4  3. 5 or 6  4. 7 to 9  5. 10 or more |
| Alcohol-related violence | As a result of your drinking, have you and others been involved in violent actions and aggression? | 1. No  2. Yes, but not in the las 12 months  3. Yes, during the last 12 months | 1. No  2. Yes, but not in the las 12 months  3. Yes, during the last 12 months |
| Smoke cannabis | Cannabis (dagga, marijuana, pot, grass, hash, etc.) | 1. Never  2. once or twice  3. Monthly  4. Weekly  5. Almost daily | 1. Never  2. once or twice  3. Monthly  4. Weekly  5. Almost daily |
| Share injections | Have you ever shared needle injections? | 1. No, never  2. Yes, in the past 3 months  3. Yes, but not in the past 3 months | 1. No, never  2. Yes, in the past 3 months  3. Yes, but not in the past 3 months |
| Sex of most recent sexual partner | Is your partner a male or a female? Most recent person with whom you had sex | 1. Male  2. Female | 1. Male  2. Female |
| Type of most recentt sex | What type of sex do you have with your partner? Most recent person | Vaginal 1. Yes 2. N0  Oral 1. Yes 2. No  Anal sex 1. Yes 2. No | Vaginal 1. Yes 2. N0  Oral 1. Yes 2. No  Anal sex 1. Yes 2. No |
| Still sexually active with partner | Are you still sexually active with your partner? Most recent person | 1. Yes  2. No | 1. Yes  2. No |
| Frequency of sexual intercouse within the last 30 days | How many times during the last 30 days did you have penetrative sexual intercourse - Most recent person | Integers | 1. < 5  2. 2- 9  3. 10 – 19  4. 20 or more |

**Table S6. Variable Definition: Exposure Variables (Health status/ Pre-existing medical conditions/ disabilities/ Stigma/ Violence)**

| **Variable Name** | **Variables** | **Variable Descriptions** | **Variable Recode** |
| --- | --- | --- | --- |
| Disability status | Whether the respondent has a disability | 1. Yes  2. No  3. Don’t know | 1. Yes  2. No  3. Don’t know |
| Duration of disability | How long has the respondent had a disability in years | Integers | 1. < 1 year  2. 2-4 years  3. 5 - 7 years  4. > 7 years |
| Type of disability | What is the disability? | 1. Physical (spinal injury, loss of a limb, etc.)  2. Sight  3. Partial hearing  4. communication/ speech  5. Mental or psychiatric illness | 1. Physical (spinal injury, loss of a limb, etc.)  2. Sight  3. Partial hearing  4. communication/ speech  5. Mental or illness |
| Ever had TB | Have you ever been told by a doctor or other health professional that you had TB? | 1. Yes  2. No  3. Don’t know | 1. Yes  2. No  3. Don’t know |
| Stigmatized due to TB | When you fell sick with TB, were you - Teased, insulted or sworn at | 1. Yes  2. No | 1. Yes  2. No |
| Vaginal discharge within the last 12 months | During the last 12 months have you had an abnormal discharge from your vagina? | 1. Yes  2. No  3. do not know | 1. Yes  2. No  3. do not know |
| Vaginal ulcer or sore within the last 12 months | During the last 12 months, have you had an ulcer or sore on or near your vagina? | 1. Yes  2. No  3. do not know | 1. Yes  2. No  3. do not know |
| Discharge from the penis within the last 12 months | During the last 12 months have you had an abnormal discharge from your penis? | 1. Yes  2. No  3. do not know | 1. Yes  2. No  3. do not know |
| Ulcer or sore near the penis within the last 12 months | During the last 12 months, have you had an ulcer or sore on or near your penis? | 1. Yes  2. No  3. do not know | 1. Yes  2. No  3. do not know |
| Experienced pain when passing urine | During the last 12 months have you experienced pain when passing urine? | 1. Yes  2. No  3. do not know | 1. Yes  2. No  3. do not know |
| Visist health facility | Did you visit a health facility or see a healthcare provider because of these problems? | 1. Yes  2. No | 1. Yes  2. No |
| Obtained treatment | Did you get treatment because of these problems? | 1. Yes  2. No | 1. Yes  2. No |
| Pregnancy status within the last 24 months | Have you been pregnant in the last 24 months? | 1. Yes  2. No | 1. Yes  2. No |
| Current pregnancy status | Are you pregnant now? | 1. Yes  2. No | 1. Yes  2. No |
| Visist clinic during current pregnancy | Have you visited an antenatal clinic during this pregnancy? | 1. Yes  2. No | 1. Yes  2. No |
| Offered HIV test | During this current pregnancy, was an HIV test offered to you during any of your antenatal care clinic visits? | 1. Yes  2. No  3. Do not know | 1. Yes  2. No  3. Do not know |
| Tested during current pregnancy | During this current pregnancy, have you been tested for HIV during any of your antenatal care clinic visits? | 1. Yes  2. No | 1. Yes  2. No |
| General wellbeing | In general, would you say that your health is excellent, good, fair or poor? | 1. Excellent  2. Good  3. Fair  4. Poor | 1. Excellent  2. Good  3. Fair  4. Poor |
| Hypertension | Hypertension/high blood pressure - Diagnosed with illness | 1. Yes  2. No  3. Refused to answer | 1. Yes  2. No  3. Refused to answer |
| Sexual violence by partner | Did your partner ever - Physically force you to have sexual intercourse with him/her | 1. Yes  2. No | 1. Yes  2. No |

**Table S7. Variable Definition: Exposure Variables (Knowledge, Awareness and Perception of HIV/AIDS)**

| **Variable Code/ Name** | **Variables** | **Variable Descriptions** | **Variable Recode** |
| --- | --- | --- | --- |
| Knowledge - HIV prevention | Whether respondent knows ways HIV can be prevented | 1. It can’t be prevented  2. Using condoms  3. Sticking to one sex partner  4. Being faithful to one sex partner who is also faithful to you  5. Reducing number of sex partners  6. Abstaining from sex  7. Avoiding contact with blood  8. Using drugs to prevent HIV transmission from mother to child  9. Medical male circumcision (as an HIV prevention method)  10. Microbicides (gel/ring inserted into the vagina to prevent HIV infection)  11. PrEP (taking ARVs to prevent HIV infection)  12. Other  13. I don’t know | 1. It can’t be prevented  2. Using condoms  3. Sticking to one sex partner  4. Being faithful to one sex partner who is also faithful to you  5. Reducing number of sex partners  6. Abstaining from sex  7. Avoiding contact with blood  8. Using drugs to prevent HIV transmission from mother to child  9. Medical male circumcision (as an HIV prevention method)  10. Microbicides (gel/ring inserted into the vagina to prevent HIV infection)  11. PrEP (taking ARVs to prevent HIV infection)  12. Other  13. I don’t know |
| Knowledge - AIDS | Can AIDS be cured? | 1. Yes  2. No  3. I don’t know | 1. Yes  2. No  3. I don’t know |
| Knowledge – HIV risk | Can a person reduce the risk of HIV by having fewer sexual partners? | 1. Yes  2. No  3. I don’t know | 1. Yes  2. No  3. I don’t know |
| Knowledge – HIV acquisition | Can a healthy-looking person have HIV? | 1. Yes  2. No  3. I don’t know | 1. Yes  2. No  3. I don’t know |
| Knowledge - PMTCT | Can HIV be transmitted from a mother to her unborn baby? | 1. Yes  2. No  3. I don’t know | 1. Yes  2. No  3. I don’t know |
| Knowledge – HIV transmission | Can the risk of HIV transmission be reduced by having sex with only one uninfected partner who has no other partners? | 1. Yes  2. No  3. I don’t know | 1. Yes  2. No  3. I don’t know |
| Knowledge – HIV transmission | Can a person get HIV by sharing food with someone who is infected? | 1. Yes  2. No  3. I don’t know | 1. Yes  2. No  3. I don’t know |
| Knowledge – HIV prevention by condom use | Can a person reduce the risk of getting HIV by using a condom every time he/she has sex? | 1. Yes  2. No  3. I don’t know | 1. Yes  2. No  3. I don’t know |
| Knowledge – HIV prevention by male circumcision | Can medical male circumcision reduce the risk of HIV infection in males? | 1. Yes  2. No  3. I don’t know | 1. Yes  2. No  3. I don’t know |
| Knowledge – HIV prevention by ART | Can the risk of HIV transmission through sex be reduced by a HIV-positive partner consistently taking drugs that treat HIV? | 1. Yes  2. No  3. I don’t know | 1. Yes  2. No  3. I don’t know |
| Knowledge – PMTCT | Can a woman infected with HIV have an HIV negative baby? | 1. Yes  2. No  3. I don’t know | 1. Yes  2. No  3. I don’t know |
| Awareness of HIV treatment | Is there a treatment for HIV/AIDS? | 1) Yes  2) No  3) I don’t know | 1) Yes  2) No  3) I don’t know |
| Knowledge of HIVtreamtment duration | How long do people have to stay on that treatment for HIV/AIDS? | 1. For the rest of their lives  2. As long as they want  3. Until they feel better  4. Until they are cured  5. Do not know  6. Other (specify) | 1. For the rest of their lives  2. As long as they want  3. Until they feel better  4. Until they are cured  5. Do not know  6. Other (specify) |
| Know a place to get an HIV test | Whether respondents know a place where they can get an HIV test | 1) Yes  2) No | 1) Yes  2) No |
| Perception of politican influence in HIV control | Political leaders are committed to controlling HIV/AIDS in South Africa | 1. Agree  2. Disagree  3. Do not know | 1. Agree  2. Disagree  3. Do not know |
| Perception of Government Support of HIV | The government supports people and families living with HIV/AIDS | 1. Agree  2. Disagree  3. Do not know | 1. Agree  2. Disagree  3. Do not know |
| Perceived risk of becoming infected with HIV | On a scale of 1 to 4 (with 1 being low and 4 being high), how would you rate yourself in terms of risk of becoming infected with HIV? | 4. You are definitely going to get infected with HIV  3. You are probably going to get infected  2. You probably won’t get infected  1. You definitely will not get infected with HIV  9. Already HIV positive | 4. You are definitely going to get infected with HIV  3. You are probably going to get infected  2. You probably won’t get infected  1. You definitely will not get infected with HIV  9. Already HIV positive |
