# Supplementary material for: The Application of Machine Learning Algorithms to Predict HIV Testing in Repeated Adult Population–Based Surveys in South Africa: Protocol for a Multiwave Cross-Sectional Analysis
Source: JMIR Res Protoc. 2025 Jan 27;14:e59916. doi: 10.2196/59916 (PMC11811654; doi:10.2196/59916)
Supplement: Multimedia Appendix 9 [file resprot_v14i1e59916_app9.pdf]

FACULTY OF HEALTH SCIENCES
RESEARCH ETHICS COMMITTEE

NHREC Registration: REC 241112-035

ETHICAL CLEARANCE LETTER
(RECX 2.0)

Student/Researcher Name	Musa Jaiteh	Student Number	223242471
Supervisor Name	Phaswana-Mafuya, Metse		
Department	Environmental Health		
Research Title	INTEGRATION OF MACHINE LEARNING ALGORITHMS TO PREDICT HIV TESTING ASSOCIATIONS USING REPEATED CROSS-SECTIONAL SURVEY DATA IN AN ADULT SOUTH AFRICAN POPULATION: AN HIV TESTING PREDICTIVE MODEL		
Date	23 April 2024	Clearance Number	**REC-2725-2024**

Approval of the research proposal with details given above is granted, subject to any conditions under 1 below, and is valid until 2025/04/22.

1. Conditions:
Gatekeeper permission, as required.
**Please note that failure to comply with the conditions above (if any) prior to implementation of the research will invalidate this ethical clearance.*

2. Renewal:
It is required that this ethical clearance is renewed annually, within two weeks of the date indicated above. Renewal must be done using the Ethical Clearance Renewal Form (REC 10.0), to be completed and submitted to the Faculty Administration office. *See Section 12 of the REC Standard Operating Procedures*.

3. Amendments:
Any envisaged amendments to the research proposal that has been granted ethical clearance must be submitted to the REC using the Research Proposal Amendment Application Form (REC 8.0) prior to the research being amended. Amendments to research may only be carried out once a new ethical clearance letter is issued. *See Section 13 of the REC Standard Operating Procedures*.

4. Adverse Events, Deviations or Non-compliance:
Adverse events, research proposal deviations or non-compliance must be reported within the stipulated time-frames using the Adverse Event Reporting Form (REC 9.0). *See Section 14 of the REC Standard Operating Procedures*.

The REC wishes you all the best for your studies.

Yours sincerely.

Prof. Christopher Stein
Chairperson: REC
Tel: 011 559 6564
Email: cstein@uj.ac.za

Secretariat: Ms Raihaanah Pieterse
Tel: 011 559 6073 email: rpieterse@uj.ac.za
